# The Clinical Epidemiology of Spontaneous ICH in a Sub-Sahara African Country in the CT Scan Era: A Neurosurgical In-Hospital Cross-Sectional Survey

**DOI:** 10.3389/fneur.2015.00169

**Published:** 2015-08-05

**Authors:** Amos Olufemi Adeleye, Uyiosa A. Osazuwa, Godwin I. Ogbole

**Affiliations:** ^1^Department of Surgery, Division of Neurological Surgery, College of Medicine, University College Hospital (UCH), University of Ibadan, Ibadan, Nigeria; ^2^Department of Neurological Surgery, University College Hospital (UCH), Ibadan, Nigeria; ^3^Department of Radiology, College of Medicine, University College Hospital (UCH), University of Ibadan, Ibadan, Nigeria

**Keywords:** spontaneous intracerebral hemorrhage, clinical epidemiology, CT pattern, sub-Saharan Africa

## Abstract

**Background:**

There is paucity of data-driven scientific reports from sub-Saharan Africa on the burden of spontaneous intracerebral hemorrhage (sICH). We have maintained a prospective consecutive in-hospital database of cases of sICH referred for neurosurgical intervention over a 5-year period.

**Methods:**

This is a cross-sectional descriptive study of the clinical epidemiology and brain computed tomography (CT) characterization of sICH from the database in this region in the current era.

**Results:**

There were 63 subjects, 38 (60.3%) males, aged 28–85 years, mean 55.7 (SD, 12.7), the modal age distribution being the sixth decade. Uncontrolled hypertension was the main predisposition in the study: present, premorbid, in 79%, but uncontrolled in 88% of these known cases, and exhibited malignant derangements of blood pressure in more than half. The clinical ictus to in-hospital presentation was delayed, median 72 h; was in severe clinical state in 70%, 57% was comatose; and was complicated with fever in 57% and respiratory morbidity in 55.6%. The main clinical symptomatology was hemiparesis, headache, vomiting, and aphasia. The sICH was supratentorial on brain CT in 90.5%, ganglionic in 50.8%, and thalamic in 58.3% of the latter. The bleed had CT evidence of mass effect and intraventricular extension (IVH) in more than half. Twenty-three patients (36.5%) underwent operative interventions.

**Conclusion:**

In this patient population, sICH is mainly ganglionic and thalamic in location with significant rate of associated IVH. In-hospital clinical presentation is delayed, and in a critical state, the bleeding is uncontrolled hypertension related in >95%.

## Introduction

Primary or spontaneous intracerebral hemorrhage (sICH) is the most severe form of cerebrovascular accidents (CVA) and the one with the highest case fatality rate (1, 2). Although there is a general dearth of data-driven scientific studies characterizing its clinical epidemiology in these regions, anecdotal reports and some expert opinions suggest that the incidence and frequency of sICH in cases of stroke are burgeoning in the low- and middle-income countries (LMIC) of the world, including sub-Saharan Africa (SSA), and are now much higher than in the west (3–7). Two recent reports further show that there is a rise in the global population prevalence of strokes as well as the mortality and significant morbidity rates in general. As of 2010, it was noted that the global burden of stroke is very asymmetrically distributed between the high-income countries (HIC) and the LMIC. It was much higher in the latter (8). Stroke in the developing world causes more than 70% of the global stroke deaths and loss of almost 80% of the disability-adjusted life years (DALY) (8, 9). Also, the LMIC contribute about 80–90% of the global stroke burden in children (aged <20 years old) and young and middle-aged adults (20–64 years) (8, 9).

In short, it has also been shown, even more poignantly in economic terms, that a country’s gross domestic product (GDP) correlates inversely with the proportion of sICH. Thus, lower GDP and health expenditures, as is the case in most LMIC, were associated with higher incidence of strokes, case fatality, proportion of hemorrhagic stroke, and lower age at stroke onset (10).

It is to be noted particularly that most of the literature on sICH from SSA have been hospital-based studies in which all categories of stroke are grouped together as one disease entity (11–16). Many of these reports often mention only sICH in passing among their stroke cohorts and most usually used only clinical evaluations sans imaging with brain computed tomography (CT) to characterize their stroke patients (14, 17).

In this light, we have recently developed a prospective consecutive database of sICH referred for possible operative interventions in our academic neurosurgical practice in a sub-Sahara African country. In this report, the findings of a cross-sectional survey of the clinical epidemiology of these sICH cases acquired over a 5-year period are presented. We also, perhaps for the first time in an indigenous patient population, characterize the pattern and distribution on brain CT of sICH in an SSA country in the current era.

## Materials and Methods

This is a cross-sectional descriptive study of the clinical epidemiology of sICH cases managed by an academic neurosurgeon in a sub-Sahara African university teaching hospital. Data collection for the study spanned a 5-year period – December 2009 till December 2014. Academic neurological surgery in this hospital is practised by a four-member faculty, each taking care of clinical cases referred to them. The subjects of this survey were only the cases of sICH that were referred by other medical personnel for neurosurgical evaluation and management. These other medical personnel were usually neurologists and/or other physicians in and out of our own university teaching hospital. The clinical records of all the cases of sICH, managed personally by the principal author over this time period, had been captured prospectively and consecutively using predesigned questionnaires and electronic spreadsheet.

All the subjects with sICH were ultimately characterized with brain CT. The clinical information extracted for this in-hospital descriptive epidemiological survey included some demographics; clinical presentation, including presence of possible co-morbidity/causation like hypertension, diabetes, and so on; the duration of hypertension or diabetes and the type of treatment received; and presence as well as the pattern of the intracerebral bleed on the CT. The ICH was deemed hypertension related on clinical presentation if there was a background history of hypertension in the subjects. In those without prior history, hypertension on initial evaluation needing medical control in the first few days of admission was also concluded the cause of the bleed, especially in the absence of any other known clinical predisposition to non-traumatic ICH. In those with hypertension, the derangement in blood pressure (BP) at admission and the first few days into the commencement of therapy was further classified empirically as mild if the BP readings ranged between 150/90 and 184/104; moderate if 185/105 and 209/114; and malignant if >210/115. We have also attempted an analysis of the in-hospital clinical outcome of the care of this cohort of patients. The findings of this latter analysis shall be the subject of a separate report.

On receipt of each patient following referral, a decision for or against operative neurosurgical intervention is usually made based on the patients’ clinical and radiological (CT) characteristics. Patients who presented in coma and had intraventricular extension (IVH) of their sICH were usually treated non-operatively, whereas the surgical group was usually those with more superficial and larger bleeds with mass effect on CT. The surgical patients also more usually showed clinical features of herniation like anisocoria. Operative interventions are usually placements of external ventricular drains or craniotomy with evacuations of the bleed. The default technique for the latter in the hands of this neurosurgeon is a mini-craniotomy under local anesthesia (with or without sedation). Cases not operated are admitted in the hospital intensive care unit or on the clinical wards. They are given isotonic fluids, antihypertensives, hypoglycemic agents as necessary, and physical therapy including thrombo-embolic deterrent (TED) stockings. Apart from other indicated supportive care like antipyretics, analgesics, and anticonvulsants, they are not given antiplatelets therapy as a rule.

### Statistical analysis

The data were analyzed with a commercial software, the SPSS version 21 (SPSS Inc., IL, USA). The results are presented in texts and descriptive statistics as sizes, frequencies and proportions, and in tabular forms. The Student’s *t-*test was used to compare means of continuous parametric variables, the Mann–Whitney *U* test to compare medians of non-parametric continuous variables, and comparisons of proportions with the Chi-square or Fisher’s exact test. An alpha value <0.05 was deemed statistically significant for associations.

## Results

Sixty-three patients were studied, 38 of them were males (60.3%) and 25 (39.7%) females. The age distribution showed 1 person (1.6%) to be below 30 years of age; 8 (12.7%) in the fourth decade (31–40 years) of life; 12 (19%) in the fifth decade; 21 (33.3%) in the sixth; 15 (23.8%) in the seventh; 3 (4.8%) in the eighth, and another 3 (4.8%) in the ninth decade. This shows the modal age distribution (33.3%) to be in the sixth decade of life, and also that as much as two-thirds of the cases are in the sixth decade and beyond. The mean ages of the males and females were similar, males versus females, 54.18 (10.40) versus 58.00 (15.52), *p* = 0.24, 95% confidence interval (CI) −10.34 to 2.71.

### Clinical predisposition to sICH

Uncontrolled hypertension at the initial clinical evaluation was the main predisposition to sICH in these study subjects (Table 1). It was a known premorbid condition in 50 of 63 cases (79.4%), and was discovered only on the index admission for the sICH in another 10 (15.9%). It was present premorbid for a range of 2 months to 30 years (median, 4.0 years), and 44 (88.2%) of the 50 patients and/or their relations averred non-compliance with the anti-hypertension drug treatment. Overall, the sICH was deemed hypertension related in as much as 95.2% (60/63 cases) in this study. Also, derangements in BP readings were documented and deemed based on our classification in the Section “Materials and Methods” above, as mild in 9 cases (14.2%), moderate in 18 (28.6%), and malignant in 33 (52.4%).

**Table 1:** **Hypertension as a predisposition to sICH in this study**.

Variables	No (%)
Premorbid history of hypertension
Unknown	13 (20.6)
Known	50 (79.4)
Compliant on antihypertensive
Yes	6 (11.8)
No	44 (88.2)
Admission BP within control (≤140/90 mmHg): *N* = 61*
Yes	10 (16.4)
No	51 (83.6)
BP derangements during treatment: *N* = 60^a^
Mild (150/90–184/104)	9 (14.2)
Moderate (185/105–209/114)	18 (28.6)
Malignant (>210/115)	33 (52.4)

*Information not available for some cases.

The other possible risk factors for developing sICH among this study population were diabetes mellitus (DM) in five (7.9%) and previous history of transient ischemic attack (TIA) or established CVA in eight cases (12.7%). Five cases (7.9%) reported the usage of aspirin before and up to the time of the ictus, but no specific information about the dosage was available on our review. There was a family history of hypertension, CVA, and DM in 14 (22.2%), 6 (9.5%), and 3 (4.8%), respectively.

### Clinical presentation

Clinical presentation for neurosurgical care was emergency in 98.4% (62/63), 35 (57.2%) presenting in coma, Glasgow coma score (GCS) <8/15. The mean duration of ictus to neurosurgical presentation was 89.68 h (SD, 89.23); the median, 72 h (range 3–456 h). Only five patients (8.2%) presented within 6 h of ictus, 29.5% within the first 24 h, and as much as 44.3% after 72 h.

The other presenting complaints included hemiparesis, headache, vomiting, aphasia, seizures, and visual impairments, in the order of frequency (Table 2). Complications of the ictus included high fever (temperature >38.5°C), and dyspnea with and without clinical evidence of aspiration pneumonitis. The presence of high fever had significant association with that of respiratory distress/aspiration pneumonitis on admission (chi-square 21.723, df^2^, *p* < 0.001). High fever was also more likely in the comatose patients (GCS <8/15) than those more lucid (chi-square 23.54, df^2^, *p* < 0.001). Other clinical complications of the sICH on admission were meningism in 23 (36.5%) and clinical evidence of deep vein thrombosis in 1 patient. The patients were adjudged critically ill in 44 (69.8%) cases.

**Table 2:** **Clinical presentation of sICH in a sub-Sahara African patient cohort in the CT era**.

Variable	*N* (%)
Level of consciousness at presentation	
Lucid	5 (7.9)
Altered sensorium	22 (34.9)
Coma (GCS <8/15)	36 (57.2)
Clinical symptomatology	
Limb paresis	39 (61.9)
Headache	39 (60.3)
Vomiting	32 (50.8)
Aphasia	27 (42.9)
Seizures	14 (22.2)
Visual impairment	9 (14.2)
Clinical complications of ictus at admission	
Fever (temperature >38.50°C)	36 (57.1)
Respiratory distress	12 (19.1)
Aspiration pneumonitis	23 (36.5)

### Pattern of sICH on CT scan in the study subjects

**Figure 1:**
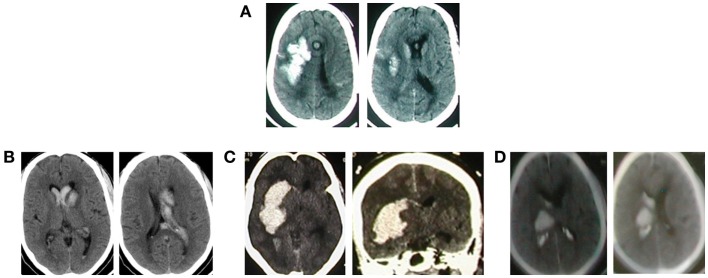
**(A)** Primary ICH in an 85-year-old woman. Deep cortical/white matter distribution and some staining of the subarachnoid space. There is local mass effect; **(B)** left caudate nucleus bleed with intraventricular extension; **(C)** right 50 mm by 60 mm putaminal bleed with intraventricular extension; **(D)** a right thalamic bleed with intraventricular extension.

**Figure 2:**
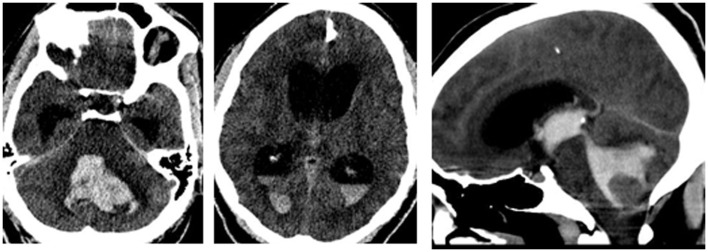
**A left cerebellar sICH with intraventricular extension**. The sagittal reconstruction image shows the hematoma casting the IV ventricle and aqueduct of Sylvius.

All the 63 cases had brain CT diagnosis of the sICH, but the median time from ictus to acquisition of this imaging was 1 day (range 1–11), or mean 2.67 days (SD, 2.67). Table 3 and Figures 1 and 2 show a spectrum of some of the findings. All had non-trauma-related intracranial bleeding on their CT, 56 (88.9%) of them purely intra-axial. Further analysis revealed no significant associations (chi-square 7.28, *p* = 0.12) between the different age groups of the study subjects (including those >60 years of age) and the intraparenchymal location of the sICH. The rest was a mixture of intracerebral and extra-axial bleed. Six of these were intracerebral with associated subarachnoid extension (Figure 1A). The ICH was mostly ganglionic in this series and mainly thalamic (Table 3). The hemorrhage was severe in the majority: mean ICH diameter on the CT 50.0 mm (SD, 18.9); median volume (using the ABC/2 rule) was 28.00 ml (range 1–132 ml); there was CT evidence of mass effect (ventricular effacement/midline shift) in 47 cases (78.3%), and ventricular extension of bleed in 41 (65.1%) (Figure 1).

**Table 3:** **Computed tomographic classification of sICH in this sub-Sahara African neurosurgical series**.

Variable	*N* (%)
Tentorial location of bleed	
Supratentorial	57 (90.5)
Infratentorial	5* (7.9)
Supra – with infratentorial component	1 (1.6)
Distribution of the supratentorial bleed	
Superficial lobar	11 (17.5)
Deep lobar/white matter	15 (23.8)
Ganglionic	32 (50.8)
Ganglionic bleed	
Thalamic	21 (58.3)
Putamen	10 (27.8)
Caudate	5 (13.9)
Laterality of bleed	
Right	29 (47.5)
Left	28 (45.9)
Bilateral	4 (6.6)

*One patient had supratentorial ICH with infratentorial component.

### Definitive care received

Based on the clinical and CT characteristics, 23 patients (36.5%) had neurosurgical operative intervention, mainly mini-craniotomy and evacuation of the hematoma. A few had EVD placement. The rest had non-operative care. The surgical group had a shorter duration of presentation for neurosurgical care than the non-surgical one, median 23.26 versus 33.72 h (Mann–Whitney *U* 257.50, *p* < 0.03). In all, the clinical in-hospital outcome was comparable between the two treatment arms. The details of this pattern of outcome and their determinants have been presented in a companion work (unpublished yet, but under review).

## Discussion

Using a prospective and consecutive database, the findings of a cross-sectional survey concerning the clinical epidemiology of spontaneous, or primary, intracerebral hemorrhage presenting for neurosurgical care in a sub-Sahara African country in the current era are presented here. This is perhaps the first study from this African sub-continent with a 100% CT rate for the management of sICH. A quick point to note, however, is the fact that there may be a possible selection bias in the patient cohort, as the referring primary care physicians, in most cases neurologists, had previously determined those most likely to benefit from neurosurgical intervention.

People in the fifth to seventh decades bear the brunt of the burden of sICH in this SSA series. Uncontrolled hypertension was malignant in more than half of the cases. The clinical presentation was severe and delayed, median time to neurosurgical attention being 72 h. Finally, the intracranial distribution of primary ICH in this study was nearly in the ratio of 7:1 for supratentorial: infratentorial compartments; the hemorrhage was purely intra-axial in 89%, and most commonly in the thalamus and the rest of the basal ganglia. And most probably following from the latter point, the sICH was complicated with IVH, a poor prognostic sign, in as much as 65%.

### Study limitations

The clinical epidemiology of stroke is best characterized by community/population surveys (11, 13, 18) not a hospital-based clinical series like this and most other documents on stroke from the LMIC (4, 7, 19). The fact that it is also only a single-physician, probably highly selected, clinical in-hospital cohort makes it impractical to make population generalizations from its findings. It may, however, be said to be the only such avenue possible to catch a glimpse of the in-hospital clinical epidemiology of patients with this severe form of stroke, sICH, in this region. There is also an unselective prospective bent to the data capture, a high CT rate, and a more definitive ante-mortem characterization of the presence and distribution of primary ICH for the first time in this indigenous patient population.

### Stroke in SSA

There is paucity of original scientific reports on stroke in general from most LMIC of SSA (5, 19). Even so, most of the few studies on this subject report just on the burdens of cerebrovascular diseases in general (12, 14, 17, 20–23). These studies and some other systematic reviews available show that stroke has now not only ceased to be the non-existent disease that it was hitherto presumed to be among indigenous black populations but also that its incidence has actually now reached an epidemic level in the LMIC (1, 3, 4). Its victims are somewhat younger by about two decades than those in the West (3). There is a fast rising level of BP in SSA and stroke is hypertension-related in the majority, even the leading cause of hypertension-related complications in some populations (2, 5, 7, 24). Furthermore, the prevalence of severe and disabling stroke is reported to be much higher in developing countries leading to much worse stroke case fatality rates (1, 4–7, 16). Although this study was specifically on primary ICH, the findings are in agreement, in many instances, with some of these established grim facts of the epidemiology of stroke in LMIC in general, and the SSA in particular. This, therefore, provides in the LMIC areas for primary prevention through increased hypertension screening and regular community-based follow-up for compliance with the drug treatment of hypertension. It also calls for community education about emergent presentation to the hospital when stroke symptoms are present for appropriate early management. The last point cannot be overemphasized in the developing world. As of 2010, it was noted that most of the global burden of stroke resides in the developing world, causing more than 70% of the global stroke deaths and loss of almost 80% of the DALY (8, 9). Also, LMIC contributes about 80–90% proportion of the global stroke burden in children (aged <20 years old) and the young and middle-aged adults (20–64 years) (7–9). The implications of this burden of disease and disability on the people in the working age for the economy of this impoverished region (LMIC) of the world need no gain saying. Thus, there is a need for innovative, more actionable measures to curtail the burgeoning burden of strokes therein.

### Pattern of sICH in SSA

There is even a greater paucity of data-driven scientific reports on the pattern of sICH in LMIC. We are aware of only one or so (25). Yet, there is increasing empirical evidence that the incidence and frequency of sICH in these regions are much higher than in the west; 29–60% versus 16–20% (4, 5, 19). The differences in the mortality rates between the two economic regions of the world are also staggering, average 28% in the west versus 37–94% in developing countries (2, 7, 19). Some of the findings of this study suggest why this may be so. The patients presented late for definitive care and had clinical and radiological evidence of severe complicated disease.

The ante-mortem documentation of the intracranial pattern of primary ICH for an SSA population shown in this study is also a unique one. Due to the well-known socio-economic limitations of health care in the LMIC, most extant scientific reports on stroke from SSA are not brain CT controlled (11, 14, 17, 18). Characterization of CVA in ischemic or hemorrhagic was usually based only on clinical judgments, which are sometimes supplemented with some eponymous clinical scoring systems, such as Siriraj Stroke Score (15). The few CT-controlled reports on CVA in general in the SSA usually record a low CT rate for the patient cohorts (15), find sICH rates of 28–52% among their patients population (15, 16, 20, 26, 27), and usually fail to describe the intracranial distribution of the hemorrhage. The only other report known to us essaying, but not quite successfully at that, to specifically characterize the intracranial distribution of primary ICH from an indigenous SSA population was a post-mortem, not a CT-based study, and is somewhat dated (21). It only showed the distribution of the ICH as “right and left cerebrum” in 43% each, pons in 3.8%, cerebellum in 3.1%, and so on.

## Conclusion

In this in-hospital series from a neurosurgical unit in the SSA, a clinical characterization of the epidemiology of sICH shows it to be a disease of people mainly in the sixth decade of life, and affecting male and female in the ratio of 1.5:1. The main predisposition is uncontrolled hypertension in up to 95%. The clinical presentation is delayed, in severe clinical state, and is complicated with high fever and respiratory morbidities, among others, in a significant proportion. The hemorrhage on CT imaging is located mainly in the thalamus and the rest of the basal ganglia, and has poor prognostic features like significant mass effects and IVH in up to two-thirds of the patient population.

## Conflict of Interest Statement

The authors declare that the research was conducted in the absence of any commercial or financial relationships that could be construed as a potential conflict of interest.
